# Thermal Conditions and Hospital Admissions: Analysis of Longitudinal Data from Cyprus (2009–2018)

**DOI:** 10.3390/ijerph182413361

**Published:** 2021-12-18

**Authors:** Katerina Pantavou, George Giallouros, Kostas Philippopoulos, Daniele Piovani, Constantinos Cartalis, Stefanos Bonovas, Georgios K. Nikolopoulos

**Affiliations:** 1 Medical School, University of Cyprus, Nicosia 2029, Cyprus; pantavou.katerina@ucy.ac.cy (K.P.); giallouros.giorgos@ucy.ac.cy (G.G.); 2 Department of Business and Public Administration, University of Cyprus, Nicosia 1678, Cyprus; 3 Department of Environmental Physics, National and Kapodistrian University of Athens, 15784 Athens, Greece; kphilip@phys.uoa.gr (K.P.); ckartali@phys.uoa.gr (C.C.); 4 Department of Biomedical Sciences, Humanitas University, Via Rita Levi Montalcini 4, Pieve Emanuele, 20090 Milan, Italy; dpiovani@hotmail.com; 5 IRCCS Humanitas Research Hospital, Via Manzoni 56, Rozzano, 20089 Milan, Italy

**Keywords:** hospital admissions, air temperature, PET, UTCI, Cyprus, public health, health impact

## Abstract

The state of the thermal environment can affect human health and well-being. Heat stress is associated with a wide range of health outcomes increasing morbidity and mortality and is recognized as an important health risk posed by climate change. This study aims at examining the effect of thermal conditions on the daily number of hospital admissions in Cyprus. Data from eight public hospitals located in five districts of Cyprus were analyzed from 2009 to 2018. Meteorological hourly gridded data were extracted by the ERA-5 Land reanalysis database with a spatial horizontal resolution of 0.1° × 0.1°. The Physiologically Equivalent Temperature (PET) and the Universal Thermal Climate Index (UTCI) were calculated as measures of the integrated effect of meteorological variables. Negative binomial regression was fitted to examine associations between the daily number of hospital admissions and meteorological variables, PET, and UTCI. The results showed that the mean daily temperature (Tair) was positively associated with hospital admissions from any cause. Hospital admissions increased by 0.6% (*p* < 0.001) for each 1 °C increase of Tair and by 0.4% (*p* < 0.001) for each 1 °C increase of PET and UTCI. Ozone and nitrogen oxides act as confounding factors. An effect of particulate matter (less than 10 μm in diameter) was observed when the analysis focused on April to August. Thresholds above which hospital admissions are likely to increase include daily mean Tair = 26.1 °C, PET = 29 °C, and UTCI = 26 °C. Studies on heat-related health effects are necessary to monitor health patterns, raise awareness, and design adaptation and mitigation measures.

## 1. Introduction

Climate change has observable effects on all regions on Earth. Severe weather warnings are issued regularly, the global temperature has increased, rainfall patterns have changed, the water cycle has been intensified, sea levels have risen, and ocean chemistry has altered, triggering a variety of adverse impacts on human well-being and health [1]. According to the World Health Organization [2], the mortality rate is expected to increase between 2030 and 2050 due to climate change by about 250,000 additional deaths per year, from malnutrition, malaria, diarrhea, and heat stress. Social and economic factors, urbanization, population growth, and ageing may affect population vulnerability [3]. The most vulnerable areas are mainly those in developing countries with weak health infrastructure [2].

The detected increasing trend in extreme temperatures [4] poses many risks to human health. The decade 2011–2020 was the warmest on record according to the World Meteorological Organization, with the last six years being the warmest [4]. Although heat is a well-recognized weather-related risk and its effect is preventable through interventions, it remains an important cause of mortality and morbidity worldwide [2]. In the United States, heat has been identified as a leading weather-related cause of death resulting in between 0.5 and 2 deaths per million people from 1979 to 2018 [5]. In Europe, the heatwaves of 2003 and 2010 accounted for 80% of deaths due to weather-, climate-, and water-related disasters from 1970 to 2019 [6]. Heat-related morbidity is difficult to systematically monitor due to the variety and severity of the health impacts, which range from mild illness [7,8] to triggering or exacerbating cardiovascular, respiratory, and cerebrovascular diseases [9]. Most studies use archives of health services such as ambulance calls, hospital emergencies, and hospital admissions [9,10,11] to examine heat-related health effects. The main findings suggest that ambient heat may affect populations in both urban and rural areas [12], that individuals with pre-existing cardiovascular or respiratory medical conditions, with low socioeconomic status, the elderly, infants and children, pregnant women, outdoor workers and athletes are more vulnerable [13,14,15], and that thermal conditions at which health impacts may occur vary by region and climate [16,17].

In the Mediterranean region, the increase in average temperature is larger than that of the global average [18], while the Mediterranean climate (hot dry summer and mild wet winters) is related with major extreme temperature events as identified in the International Disaster Database [19]. Cyprus is an island in the eastern Mediterranean region with a warm climate of type Csa and BSh [20], and a potential future hot spot region in terms of climate change, which could be a serious threat to public health [21]. According to the census of 2011 [22], the population in the five districts of the Republic of Cyprus (government-controlled area)—Nicosia, Limassol, Larnaca, Pafos, and Ammochostos—was 840,407. Most people (38.9%) reside in the Nicosia district, which hosts Nicosia city, the capital of the island. Apart from being in a climate hot spot area, it is also affected by dust transport from the Saharan and Arabian deserts. Desert dust storms are frequently observed in Cyprus with daily particulate matter less than 10 μm in diameter (PM_10_) that can exceed 1200 μg/m^3^ [23]. Only a few studies have examined weather-related health impacts in Cyprus. They focused on mortality [21,24], PM concentrations [25], and cardiovascular and respiratory hospital admissions between 2004 and 2009 [26].

The aim of this work is to examine the effect of air temperature and outdoor thermal conditions on the daily number of hospital admissions in Cyprus between 2009 and 2018. Studies assessing and identifying health vulnerability to the prevailing thermal conditions can support state health services towards developing effective adaptation measures.

## 2. Materials and Methods

### 2.1. Hospital Admission Data

**Figure 1:**
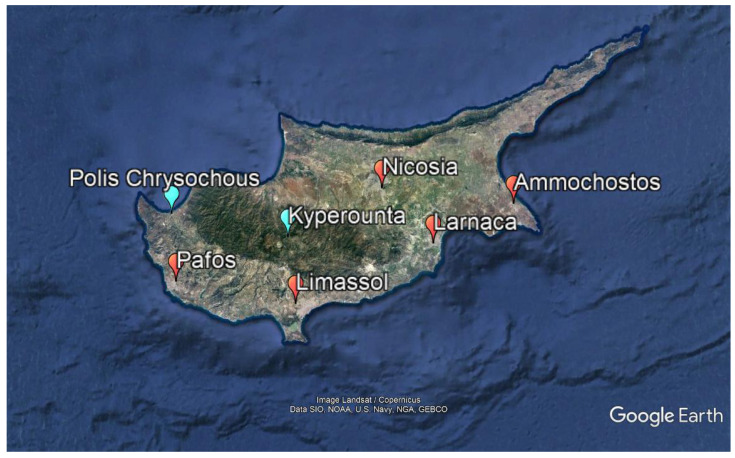
Map of Cyprus and locations of general (red dots) and rural (blue dots) hospitals. Nicosia city (Nicosia district) hosts two general hospitals (i.e., the Nicosia General Hospital and the General Hospital for Children Makario). The rural hospital of Polis Chrysochous is located in the Pafos district and of Kyperounta in the Limassol district.

Health data were provided by the Statistical Service of Cyprus [27]. The data included the daily number of hospital admissions from all causes. The data were derived from the archives of the eight public hospitals of the healthcare service of the Republic of Cyprus (Figure 1). Two general hospitals are located in the city of Nicosia (i.e., Nicosia General Hospital and General Hospital Makario); one general hospital is located in each capital of the administrative districts of Limassol, Larnaca, Pafos and Ammochostos; and two rural hospitals are located each in Polis Chrysochous (Pafos district) and Kyperounta (Limassol district). The data included information on admission date, sex, age, and diagnosis according to the International Statistical Classification of Diseases and Related Health Problems (ICD-10 Version: 2010) for the period between 1 January 2009 and 31 December 2018.

The protocol used in this study was approved by the Cyprus National Bioethics Committee (EEBE/EΠ2018/48).

### 2.2. Meteorological Data

Gridded meteorological data were extracted from the ERA-5 Land reanalysis database of the European Centre for Medium-Range Weather Forecasts (EMCWF) [28]. The ERA5-Land datasets are provided in an hourly temporal resolution and with a spatial horizontal resolution of 0.1° × 0.1°. Air temperature (Tair, °C) and dew-point temperature (Td, °C) at the height of 2 m, eastward and northward components of wind speed (WS, m/s) at the height of 10 m, as well as the cumulative amount of solar radiation reaching the Earth’s surface (SR, J/m^2^), were retrieved for the closest available grid point to the geographical location of each hospital considered in this study.

Data on air pollutant concentrations were obtained from the air quality stations in each district and closest to each hospital, from the Department of Labour Inspection of the Ministry of Labour, Welfare, and Social Insurance. Mean daily concentrations of nitrogen monoxide (NO, μg/m^3^), dioxide (NO_2_, μg/m^3^), nitrogen oxides (NO_X_, μg/m^3^), ozone (O_3_, μg/m^3^), carbon monoxide (CO, μg/m^3^), sulfur dioxide (SO_2_, μg/m^3^), and benzene (μg/m^3^) were estimated by respective hourly values. Particulate matter ≤ 10 μm in diameter (PM_10_, μg/m^3^) and ≤2.5 μm (PM_2.5_, μg/m^3^) in diameter were estimated from daily concentrations. The data were obtained from the traffic stations of Nicosia, Limassol, Larnaca, Pafos, and Paralimni, and matched to the daily number of hospital admissions to the general hospital of each district.

### 2.3. Data Process

The relative humidity (RH, %) was estimated from the Td [29,30,31], the WS was estimated at the height of 2 m in the eastward and the northward components using the logarithmic wind profile for roughness-length 1 m [32], and the SR was converted in W/m^2^. The Physiologically Equivalent Temperature (PET, °C) [33,34] and the Universal Thermal Climate Index (UTCI) [35] are thermal indices describing “the thermal environment in a thermophysiologically weighted way” [34]. They are based on the human energy balance and use several meteorological variables for the evaluation of human physiological responses. PET and UTCI are applied to all climates and are two of the most widely used indices for comparisons between different populations and climates. The calculated values of PET and UTCI in °C represent human thermal perception according to an assessment scale. The original assessment scale of PET is a nine point bipolar scale with a central neutral point (i.e., “comfortable”/“no thermal stress”, 18 °C < PET < 23 °C) ranging from “very cold”/“extreme cold stress” (i.e., PET < 4 °C) to “very hot”/“extreme heat stress” (i.e., PET > 41 °C) [36]. The UTCI original assessment scale extends to a 10-point scale ranging from “extreme cold stress” (i.e., UTCI < −40 °C) to “extreme heat stress” (i.e., UTCI > 46 °C) [37].

PET and UTCI were estimated to assess the thermal environment on an hourly basis by Tair, RH, WS, and SR using the Rayman model [38,39]. Hourly meteorological variables, PET, and UTCI were averaged daily and matched to the daily number of hospital admissions. Furthermore, hospital admissions per 100,000 population were estimated based on the population of the hospitals’ district.

### 2.4. Statistical Analysis

Statistical measures including mean, median, standard deviation, interquartile, maximum, and minimum values were used to describe the data. Spearman’s rho was used to test for trends over time.

Statistical analyses included analysis of variance (ANOVA) and negative binomial regression. The latter was used to model the response outcome (daily number of hospital admissions) against the predictive variables (meteorological and other control variables) as appropriate for count data. Negative binomial regression was chosen over Poisson regression due to the overdispersion in the distribution of daily number hospital admissions, with the mean and variance being significantly different. Negative binomial regression effectively addresses the issue of overdispersion by including a dispersion parameter that relaxes the assumption of equal mean and variance [40]. Furthermore, a random effects model was fitted to account for the cluster effects of the hospital variable [41]. The random effects model, also called a variance components model, is a kind of hierarchical model, which assumes that the data analyzed are drawn from a hierarchy of different populations whose differences relate to that hierarchy [42]. The negative binomial regression coefficient expresses the expected change in the difference of the natural logs of expected counts (natural log of the ratio of expected counts) of the response variable for one unit change in the predictor variable, given that the other predictor variables in the model are constant. The exponentiated regression coefficient is the incidence rate ratio (IRR).

The models were extended to examine the confounding effect of air quality considering all air pollutant concentrations available in our database. All models were adjusted for year and hospital. In addition, the analysis was repeated including seasonality with respect to the warm (April to November) and the cool (December to March) periods as an adjusting variable. The months were classified in warm and cool periods based on their mean values during the period of study (i.e., between 2009 to 2018, mean monthly Tair > 17 °C for the warm and Tair < 17 °C for the cold period). Moreover, seasonal sub-analysis was conducted with respect to the warm and cool period. The delayed effect of thermal conditions was examined using mean daily values averaged over 2 (0–1), 3 (0–2), and 4 (0–3) days. The analysis was carried out overall, for people older than 74 years, and for cardiovascular and respiratory causes (ICD-10 groups I and J).

Segmented piecewise linear regression was utilized to identify potential break points (thresholds) where the relationship between average daily Tair, PET, or UTCI, and hospital admissions was altered. Intervals of 0.1 °C were used to divide the data into subsets and then regression lines were fitted on subsets, modeling the association between the independent variable and the hospital admissions. The selection of breakpoint points was based on whether there was a statistically significant difference in the regression coefficients, as well as using the Akaike information criterion (AIC) and Bayesian information criterion (BIC) for evaluating competing models for best model fit [43].

The analysis was performed using the statistical software STATA 16.0 (Stata Corp., College Station, TX, USA).

## 3. Results

### 3.1. Hospital Admissions

In total, 792,984 hospital admission records were retrieved from the archives of eight public hospitals in Cyprus between 1 January 2009 and 31 December 2018 (Table 1). The median age of the hospitalized patients was 56 years (interquartile range: 27–72 years). People over 74 years old made up 21.3% (*n* = 168,803) of patients and 14.5% (*n* = 114,842) were younger than 13 years. Males were 51.5% (*n* = 408,361) of the hospitalized patients (mean age ± standard deviation: 50.3 ± 27.5 years) and 48.5% (*n* = 384,623) were females (49 ± 27 years). The admission cause was registered in 83% (*n* = 658,149) of the records. Most patients (12.5%, *n* = 82,548) were admitted for neoplasms (ICD-10: group C00-D48) following by patients (11.5%, *n* = 75,631) admitted for injury, poisoning, and other consequences of external causes (ICD-10: group S00-T98). There was no record for hospitalization cause for exposure to excessive natural heat (ICD-10: X30) or cold (ICD-10: X31), while the number of admissions for heat effects was small (*n* = 65) for conducting further analysis. The hospitalizations for cardiovascular diseases (ICD-10: group I) accounted for 10.2% (*n* = 67,382) and for respiratory diseases (ICD-10: group J) 8.9% (*n* = 58.389) of the data, respectively.

**Figure 2:**
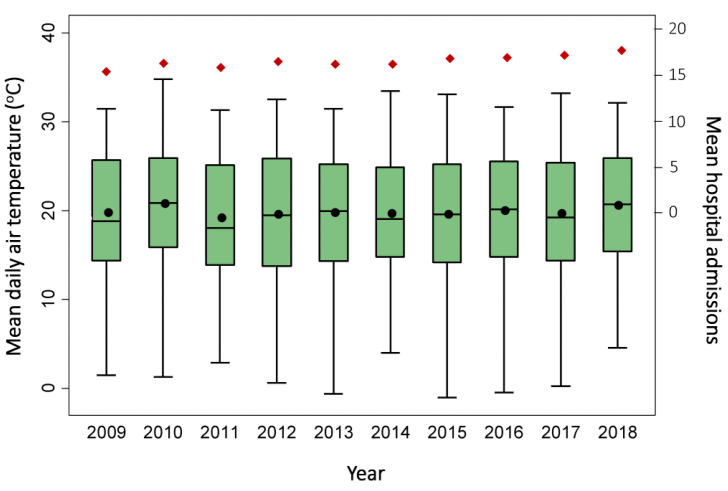
Box plot of variation of mean daily air temperature and mean hospital admissions per 100,000 population (red diamonds) in Cyprus over the years 2009–2018.

Table 1 presents the summary statistics of overall admissions, for patients over 64 years old, and for cardiovascular and respiratory diseases. The average daily number of total hospital admissions was 29.3 ± 26.1 (16.7 ± 11.3 per 100,000 population) and for cardiovascular and respiratory diseases was 2.1 ± 2 and 2 ± 1.8 per 100,000 population, respectively. The highest rate of daily mean hospital admissions (26 ± 7.8 per 100,000 population) was found in the General Hospital of Pafos (Table 1) and for cardiovascular (3 ± 1.7 per 100,000 population) and respiratory diseases (3.3 ± 2 per 100,000 population) was observed in Ammochostos. The yearly mean hospital admissions per 100,000 population ranged between 15.6 and 17.8 (Figure 2, red diamonds) with an increasing trend over the years (*p* < 0.001).

### 3.2. Meteorological and Air Quality Conditions

Table 2 summarizes the meteorological and air quality conditions between 1 January 2009 and 31 December 2018 in Cyprus. The average daily Tair was 19.9 °C with an interquartile range of 14.5–25.5 °C. The lowest value was measured in Kypernounta (16.2 ± 7.1 °C) and the highest (21 ± 5.7 °C) in Ammohostos. The median value of mean daily PET was 20.5 °C and of mean daily UTCI was 21.8 °C, both of which correspond to the category “no thermal stress” on their assessment scales. Of PET values, 21.8% were higher than 29 °C, and 35.3% of UTCI values were higher than 26 °C, both of which correspond to the threshold of “moderate heat stress” in their respective assessment scales. One-way ANOVA suggested that mean daily Tair (*p* < 0.001) differed among the years (Figure 2), showing an overall increasing trend (*p* = 0.04). An increasing trend was also observed for mean daily UTCI (*p* = 0.01). The mean daily concentration of all pollutants was below the respective European Commission guideline [44] daily or annual mean value, whereas of NO_2_ and PM_2.5_, the concentrations were over the World Health Organization air quality guideline values (25 μg/m^3^ 24 h mean for NO_2_, 15 μg/m^3^ for PM_2.5_) [45].

### 3.3. Hospital Admissions and Thermal Environment

**Figure 3:**
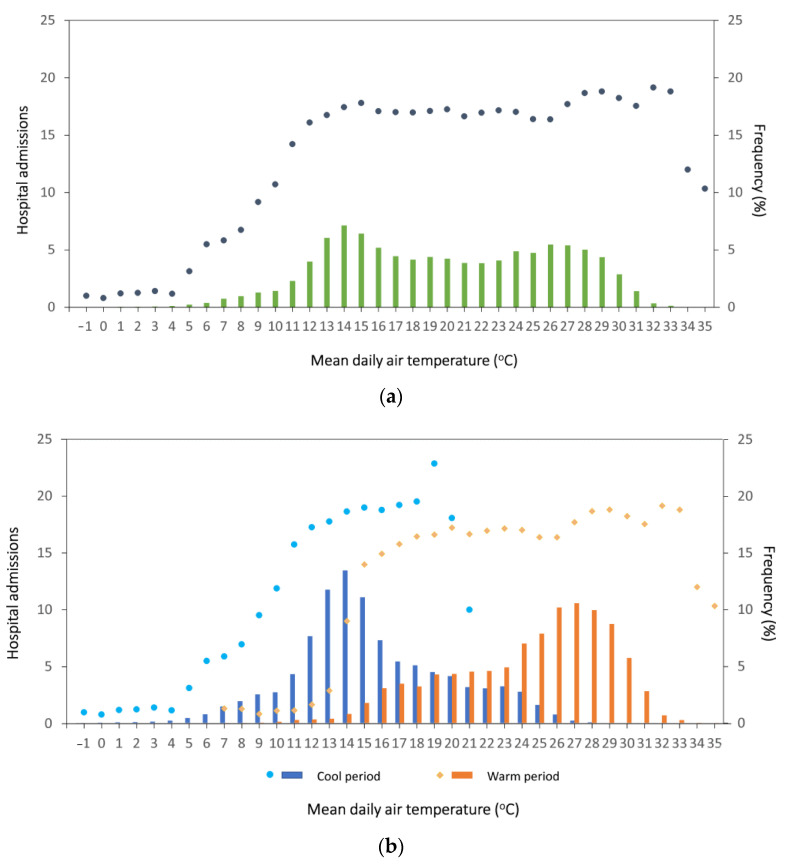
Mean hospital admissions per 100,000 population (dots and diamonds) in 1 °C intervals of air temperature in Cyprus (2009–2018) and frequency of mean daily air temperature (bars) (**a**) overall and (**b**) by cool and warm period.

Figure 3a presents the mean number of hospital admissions per 100,000 population with 1 °C intervals of mean daily Tair. The admissions ranged between 16.1 and 17.8 for air temperatures between 12 °C and 27 °C, while they were higher than 18 per 100,000 population for temperatures 28–30 °C and 32–33 °C (Figure 3a,b). Figure 3a,b shows no evidence of low temperature effects on the number of hospital admissions. Nevertheless, the frequency of days with low temperatures was relative low (7.7% for daily mean Tair < 10 °C).

Negative binomial regression models were used to examine the relationship of hospital admissions with the thermal environment. The models were developed based on Tair, PET, and UTCI while adjusting for air pollutant concentrations. The results showed that an increase of Tair, PET, or UTCI is associated with an increase of all-cause hospital admissions in Cyprus (*p* < 0.001, Models 1–3 in Table 3). The IRR was 1.006 (*p* < 0.001) in Model 1, suggesting that for each 1 °C increase of Tair, hospital admissions are expected to increase by 0.6%. This increase was 0.4% (IRR = 1.004, *p* < 0.001) for 1 °C increases of PET or UTCI. The effect of Tair, PET, or UTCI diminished for patients older than 64 years ranging from 0.2% (IRR = 1.002, *p* < 0.001) for 1 °C increase of Tair or UTCI to 0.1% (IRR = 1.001, *p* < 0.001) for 1 °C increase of PET (Models 4–6, Table 3). An increase of O_3_, NO, and NO_2_ concentrations were also associated with an increase in the number of hospital admissions. The effect of Tair, PET, and UTCI changed when the analysis focused on cardiovascular or respiratory causes, producing a negative coefficient (Models 7–10, Table 3). Τhe effects of PET and UTCI on the admissions for cardiovascular causes was not significant; therefore, they were not included in Table 3. Moreover, the effect of PM_10_ concentration was not significant in all models (Models 1–10). Similar results were found when models were additionally adjusted for warm/cool periods.

In the warm period (Table 4), the IRR were slightly higher compared to the results found considering the overall data. In Model 11, the IRR was 1.007 (*p* < 0.001), suggesting a 0.7% increase of all-cause hospital admissions for 1 °C increases of Tair. For 1 °C increases of PET and UTCI, the increase in admissions was 0.6% (*p* < 0.001; Models 12 and 13). In the cool period, the effect of Tair on admissions was not statistically significant. However, a negative effect was found for respiratory cause admissions (IRR = 0.994, *p* < 0.001; Model 24). PET and UTCI produced statistically significant associations for all-cause admissions (Models 20 and 21) and for people over 64 years (Models 22 and 23).

The effect of PM_10_ was significant when the analysis was restricted to the April–August period (Models 25–32, Table 5). Specifically, PM_10_ concentrations were positively associated with hospital admissions. The effect of thermal conditions followed the same pattern of the total period, producing positive coefficients for all-cause hospital admissions and for patients older than 64 years (Models 25–29), and negative coefficients for hospital admissions for respiratory causes (Models 30–32). NO and NO_2_ concentrations remained in the models, while the effect of O_3_ was non-significant. The effect of thermal conditions on the admissions for cardiovascular causes were not significant.

The effect of thermal conditions on hospital admissions persisted in the lag period (0–1), but the IRR decreased. One degree °C increases of Tair, PET, or UTCI (*p* < 0.001) were associated with 0.03% increases in hospital admissions.

Piecewise linear regression analysis was used to identify thresholds of Tair, PET, and UTCI above which hospital admissions increases produced interesting results. For Tair, the analysis was limited between 12 °C and 33 °C (Figure 3). Air temperatures below 12 °C were excluded because of the absence of a cool effect. Air temperatures above 33 °C were also excluded due to the low frequency of data. Piecewise regression analysis with data intervals of 0.1 °C identified 26.1 °C as a point of change. The association between Tair and hospital admissions in the range 12 °C to 26.1 °C was negative (coefficient: −0.04, *p* = 0.03), whereas for the subset 26.1 °C to 33 °C, the association was positive (coefficient: 0.2, *p* = 0.04). The difference between the two coefficients was statistically significant (*p* = 0.02).

Similarly, a PET threshold at 29 °C was identified. The piecewise regression analysis was performed for PET between 23 °C (upper threshold of “comfortable”/”no thermal stress” category of PET assessment scale) and 38 °C. The coefficients were −0.17 (*p* = 0.04) for values lower than 29 °C and 0.24 (*p* = 0.002) for values higher than 29 °C, with a statistically significant difference of 0.41 (*p* < 0.001). The UTCI threshold was estimated at 26 °C using the UTCI values between 12 °C and 40 °C. The coefficient of the relationship between hospital admissions and UTCI lower than 26 °C was −0.08 (*p* < 0.001), and for UTCI higher than 26 °C was 0.17 (*p* < 0.001). The difference between the two slopes was statistically significant (0.25, *p* < 0.001).

## 4. Discussion

This study examined the effect of thermal conditions on hospital admissions in public hospitals in Cyprus between 2009 and 2018, including all seasons and public hospitals in Cyprus. A comprehensive analysis was conducted that was not limited to the effect of air temperature, but also considered several meteorological variables and the commonly used thermophysiological indices PET and UTCI. Moreover, the confounding effect of air quality was considered.

Results showed a positive association between mean daily temperature and hospital admissions from any cause. This association was examined taking into account yearly variations and differences across hospitals, and was systematically detected independently of the measure of thermal burden, such as Tair, PET, or UTCI. O_3_, NO, and NO_2_ were found to have confounding effects. PM_10_ was also associated with hospital admissions when the analysis focused on the period between April and August. The association attenuated when the analysis was limited to the vulnerable group of people older than 64 years and became negative for admissions of cardiovascular and respiratory cause. Assuming a U-shaped association of air temperature on adverse health effects [46], there was no evidence of any low air temperature effect on hospital admissions. Nevertheless, in the cool period PET and UTCI were negatively associated with hospital admissions for any cause and for people older than 64 years. Thresholds for an increase in hospital admissions were detected for mean daily air temperature (26.1 °C), PET (29 °C), and UTCI (26 °C). The effect of thermal conditions was direct, on the same or the next day.

The results of this study agree with previously published research. Warm weather conditions were found to be associated with increased risk of morbidity reflecting an increase in emergency department visits [47], hospital admissions [10], or emergency ambulance calls [9]. In Cyprus, previous research has examined the heat-related health effects through all-cause and cardiovascular mortality [21,24,48], and cause specific (cardiovascular and respiratory) hospital admissions [26] using data up to 2010, while some studies focused only on the warm period [21,24]. Their findings support that mean daily all-cause mortality increases for daily maximum air temperatures over 32–34 °C [21]. Moreover, they suggest that for a 1 °C increase in maximum daily air temperature above 33.7 °C, the increase of relative risk for all-cause mortality is 4% [24].

The threshold of daily maximum temperature of 32 °C found in the study of Heaviside et al. [21] is in agreement with the threshold of 26 °C found in this study, given that our study focused on the daily mean instead of maximum air temperature. Moreover, the pattern of variation of mean daily mortality for each degree °C of daily maximum temperature was in line with the results of our study [21]. The thresholds of PET (29 °C) and UTCI (26 °C) found in this study are in accordance with the assessment scale of each index. PET equal to 29 °C corresponds to the threshold above which thermal sensation corresponds to the “warm” category or “moderate heat stress” grade of physiological stress. Similarly, UTCI at 26 °C is the threshold above which the stress category of UTCI is “moderate heat stress”. 

The absence of a low temperature effect on the number of hospital admissions in this analysis is in line with findings considering the effect of maximum daily air temperature on all-cause mortality in Cyprus [21]. This could be attributed to the relatively small sample of mean daily air temperatures below 12 °C, which account for 7.6% of the data. The immediate—on the same or next day—effect of thermal conditions was also found in previous studies focusing on all-cause [24] and cardiovascular [48] mortality in Cyprus, and in other areas with Mediterranean climates [49].

Daily mean air temperature (as used in this study), rather than daily maximum air temperature, has been suggested as a measure of the effect of thermal conditions on cardiovascular mortality in Cyprus [48]. It has also been found that high temperatures exacerbate cardiovascular mortality, with the highest risk for ischemic heart disease [48], and that air mass associated with warm, rainy days [23] increases hospital admissions due to cardiovascular diseases. On the other hand, negative associations between thermal conditions and cardiovascular and respiratory events during warm periods have been reported in Mediterranean climates as well [50,51]. This could be due to compliance with protection measures, increased use of air-conditioning, and reduction of physical and psychological stress in summer months as a typical long vacation period, especially for older people. Some evidence supports the finding that O_3_, PM_10_, and NOx were positively associated with all-cause and cardiovascular hospital admissions [10,25,26], whereas there are other studies reporting non-significant effects of air pollutant concentrations on morbidity [52], even in the same geographical or climate setting [24].

The temperature-related health effects, as well as their strength and changes in time, vary by the location, climate, demographic, and socioeconomic characteristics of the societies [17]. Thus, locally and climate-focused investigation of evidence should not be neglected. The findings of this study can help in understanding the impact of thermal burden on population health.

Regarding the limitations of this study, it should be noted that the hospital admission data included only individuals admitted to public hospitals in Cyprus. People experiencing mild heat-related symptoms, seeking care in hospital emergencies but who were not hospitalized, or people admitted to private hospitals were not considered. It was not possible to identify and exclude re-admissions or scheduled hospitalizations from the analysis. Nevertheless, this analysis included data from all public hospitals in Cyprus, considered both cool and warm periods, and examined the issue of thermal environment comprehensively considering thermal indices and air quality, while covering a relatively long-term period of data.

## 5. Conclusions

Exposure to heat can be a serious threat to human health. Heatwaves have caused high mortality in the past, including the 1987 heatwave in Greece, the Chicago, US heatwave in 1995, and the 2003 heatwave in Europe. Since then, information campaigns and other interventions have raised awareness and prepared people to deal with heat extremes. This study is one of a few focusing on Cyprus. It considers a relatively long time period and updated the previous evidence in the literature regarding heat-related morbidity. Warm thermal conditions seem to increase the risk for all-cause hospital admission. This estimated risk however was lower compared to previous figures. Probable reasons include individuals’ preparedness, compliance with protection and prevention measures issued by public health authorities, effective weather forecasts, and use of air conditioners that have become a necessity in home or work environments in warm climates.

Climate change is expected to increase further the intensity, duration, and frequency of heat extreme events and the number of people exposed to heat. Projection scenarios suggest a future increase in temperature and premature heat-related deaths. Although population tolerance to extreme heat may also have increased over time, awareness should be kept high and adaptation measures should be improved and updated constantly.

## Figures and Tables

**Table 1:** Hospital admissions by hospital in Cyprus between 1 January 2009 and 31 December 2018.

District	Population	Hospital	Βeds ^1^	Hospital Admissions								
				N				Daily Mean (±SD)	Daily Mean per 100,000 (±SD)			
				Total	+64 Years	Cardiovascular	Respiratory		Total	+64 Years	Cardiovascular	Respiratory
Total	840,407	All		792,984	307,615	67,382	58,389	29.3 ± 26.1	16.7 ± 11.3	4 ± 2.8	2.1 ± 2	2 ± 1.8
Nicosia	326,980	Nicosia	539	246,281 (31.1%)	111,991 (36.4%)	30,502 (45.3%)	14,388 (24.6%)	67.4 ± 27.6	20.6 ± 8.5	9.4 ± 3.9	2.6 ± 1.5	1.3 ± 0.9
		Makario	217	102,054 (12.9%)	16,375 (5.3%)	419 (0.6%)	4968 (8.5%)	27.9 ± 12	8.5 ± 3.7	1.9 ± 1	0.1 ± 0.3	0.6 ± 0.6
Limassol	235,330	Limassol	399	213,221 (26.9%)	90,097 (29.3%)	18,175 (27%)	16,421 (28.1%)	58.4 ± 16.8	24.8 ± 7.1	10.5 ± 3.8	2.2 ± 1.2	2.1 ± 1.5
		Kyperounta	ΝA	9998 (1.3%)	7321 (2.4%)	1599 (2.4%)	1220 (2.1%)	3.1 ± 1.8	1.2 ± 0.9	0.9 ± 0.8	0.2 ± 0.4	0.3 ± 0.5
Larnaca	143,192	Larnaca	191	93,931 (11.9%)	34,601 (11.3%)	7219 (10.7%)	9386 (16.1%)	25.7 ± 9.4	17.9 ± 6.6	6.5 ± 2.9	1.7 ± 1	2.2 ± 1.7
Pafos	88,276	Pafos	158	83,661 (10.6%)	31,894 (10.4%)	7258 (10.8%)	9319 (16%)	22.9 ± 6.9	26 ± 7.8	9.9 ± 3.9	2.8 ± 1.9	3.2 ± 2.3
		Polis Chrysochous	ΝA	3354 (0.4%)	2605 (0.8%)	432 (0.6%)	3319 (16%)	1.7 ± 1	1.8 ± 1.1	1.6 ± 1	1.2 ± 0.4	1.1 ± 0.4
Ammochostos	46,629	Ammochostos	97	40,484 (5.1%)	12,797 (4.2%)	1778 (2.6%)	2322 (4%)	11.1 ± 4.5	23.8 ± 9.6	7.8 ± 4.4	3 ± 1.7	3.3 ± 2

^1^ Source: www.mof.gov.cy/mof/cystat/statistics.nsf/ (accessed on 16 September 2021).

**Table 2:** Descriptive statistics of the daily means of meteorological variables, thermal indices, and air pollutant concentrations.

	Mean	Standard Deviation	Median	Interquartile Range	Minimum	Maximum
Tair (°C)	19.9	6.3	19.6	14.5–25.5	−1.0	34.8
RH (%)	68	11	69	61–76	18	97
WS (m/s)	0.9	0.4	0.8	0.6–1.0	0.1	3.5
SR (W/m^2^)	225.2	82.0	232.1	147.9–302.7	24.7	352.4
PET (°C)	20.5	8.6	20.5	12.9–28.2	−5.5	39.6
UTCI (°C)	21.7	7.8	21.8	15.0–28.6	−4.5	42.5
NO (μg/m^3^)	14.4	15.6	8.8	4.9–17.5	0.1	137.9
NO_2_ (μg/m^3^)	28.7	12.0	27.3	19.9–36.7	1.0	84.7
NO_x_ (μg/m^3^)	50.6	34.0	40.8	27.7–63.4	1.2	277.9
SO_2_ (μg/m^3^)	3.0	2.1	2.5	1.5–4.0	0.0	27.2
CO (μg/m^3^)	462.8	237.4	405.6	314.6–546.9	10.5	2130.1
O_3_ (μg/m^3^)	57.5	19.8	58.3	42.5–72.2	2.8	134.2
PM_2.5_ (μg/m^3^)	20.2	11.0	18.2	13.8–24.3	4.8	347.4
PM_10_ (μg/m^3^)	42.7	39.3	36.9	29.2–47.4	5.4	2868.2
Benzene (μg/m^3^)	1.2	0.9	1.0	0.6–1.6	0.0	29.4

**Table 3:** Negative binomial regression models for the impact of the thermal environment on the number of hospital admissions (dependent variable). All models are adjusted for admission year and hospital.

Models	Population	Independent Variable	Coefficient	IRR	*p*-Value	Lower CI	Upper CI
1	All-cause	Tair	0.006	1.006	<0.0001	1.005	1.006
		O_3_	0.001	1.001	<0.0001	1.001	1.002
		NO	0.002	1.002	<0.0001	1.001	1.002
		NO_2_	0.010	1.009	<0.0001	1.009	1.010
2		PET	0.004	1.004	<0.0001	1.003	1.005
		O_3_	0.001	1.001	<0.0001	1.001	1.002
		NO	0.002	1.002	<0.0001	1.001	1.002
		NO_2_	0.009	1.009	<0.0001	1.009	1.010
3		UTCI	0.004	1.004	<0.0001	1.003	1.005
		O_3_	0.001	1.001	<0.0001	1.001	1.002
		NO	0.002	1.002	<0.0001	1.001	1.002
		NO_2_	0.010	1.010	<0.0001	1.009	1.010
4	+64 years	Tair	0.002	1.002	0.001	1.001	1.003
		O_3_	0.001	1.001	0.000	1.001	1.002
		NO	0.001	1.001	0.028	1.000	1.001
		NO_2_	0.010	1.010	0.000	1.009	1.011
5		PET	0.001	1.001	0.001	1.001	1.002
		O_3_	0.001	1.001	0.000	1.001	1.002
		NO	0.001	1.001	0.029	1.000	1.001
		NO_2_	0.010	1.010	0.000	1.009	1.011
6		UTCI	0.002	1.002	0.002	1.001	1.002
		O_3_	0.001	1.001	0.000	1.001	1.002
		NO	0.001	1.001	0.033	1.000	1.001
		NO_2_	0.010	1.010	0.000	1.009	1.011
7	Cardiovascular diseases	Tair	−0.003	0.997	0.005	0.995	0.999
		O_3_	−0.002	0.998	<0.0001	0.997	0.999
8	Respiratory diseases	Tair	−0.025	0.975	<0.0001	0.973	0.978
		O_3_	0.002	1.001	<0.0001	1.001	1.003
		NO_2_	0.009	1.009	<0.0001	1.008	1.003
9		PET	−0.018	0.982	<0.0001	0.981	0.984
		O_3_	0.002	1.002	<0.0001	1.001	1.003
		NO_2_	0.010	1.010	<0.0001	1.008	1.011
10		UTCI	−0.018	0.982	<0.0001	0.980	0.983
		O_3_	0.017	1.002	<0.0001	1.001	1.003
		NO_2_	0.010	1.010	<0.0001	1.001	1.011

Abbreviations: CI, confidence interval; IRR, incidence rate ratio.

**Table 4:** Negative binomial regression models for the impact of the thermal environment on the number of hospital admissions (dependent variable) for the warm (April to November) and cool (December to March) period. All models are adjusted for admission year and hospital.

Population	Independent Variable	Model	Warm Period			Model	Cool Period		
			Coefficient	IRR	*p*-Value		Coefficient	IRR	*p*-Value
All-cause	Tair	11	0.007	1.007	<0.0001				
	O_3_		0.001	1.001	0.009				
	NO		0.004	1.004	<0.0001				
	NO_2_		0.008	1.008	<0.0001				
	PET	12	0.006	1.006	<0.0001	20	−0.005	0.995	0.002
	O_3_						0.005	1.005	<0.0001
	NO		0.003	1.003	<0.0001		0.002	1.002	<0.0001
	NO_2_		0.008	1.008	<0.0001		0.012	1.012	<0.0001
	UTCI	13	0.006	1.006	<0.0001	21	−0.005	0.995	0.002
	O_3_		0.001	1.001	0.009		0.005	1.005	<0.0001
	NO		0.004	1.004	<0.0001		0.002	1.002	<0.0001
	NO_2_		0.008	1.008	<0.0001		0.012	1.012	<0.0001
+64 years	Tair	14	0.002	1.002	0.013				
	O_3_		0.001	1.001	0.008				
	NO		0.002	1.002	<0.0001				
	NO_2_		0.009	1.009	<0.0001				
	PET	15	0.002	1.002	0.001	22	−0.004	0.996	0.050
	O_3_		0.001	1.001	0.018		0.004	1.004	0.000
	NO		0.002	1.002	<0.0001		0.001	1.001	0.023
	NO_2_		0.009	1.009	<0.0001		0.012	1.012	0.000
	UTCI	16	0.003	1.003	0.001	23	−0.004	0.996	0.036
	O_3_		0.001	1.001	0.008		0.004	1.004	0.000
	NO		0.002	1.002	0.000		0.001	1.001	0.023
	NO_2_		0.009	1.009	0.000		0.011	1.012	<0.0001
Respiratory diseases	Tair	17	−0.016	0.984	<0.0001	24	−0.016	0.984	<0.0001
	NO_2_		0.008	1.008	<0.0001		0.006	1.006	<0.0001
	PET	18	−0.011	0.989	<0.0001				
	NO_2_		0.008	1.008	<0.0001				
	UTCI	19	−0.012	0.988	<0.0001				
	NO_2_		0.008	1.008	<0.0001				

**Table 5:** Negative binomial regression models for the impact of the thermal environment on the number of hospital admissions (dependent variable) for April to August. All models are adjusted for admission year and hospital.

Models	Population	Independent Variable	Coefficient	IRR	*p*-Value	Lower CI	Upper CI
25	All-cause	Tair	0.007	1.007	<0.0001	1.005	1.009
		PM_10_	0.001	1.001	0.004	1.000	1.001
		NO	0.028	1.028	<0.0001	1.025	1.031
		NO_2_	0.002	1.002	0.022	1.000	1.004
26		PET	0.005	1.005	<0.0001	1.004	1.007
		PM_10_	0.001	1.001	0.002	1.000	1.001
		NO	0.008	1.028	<0.0001	1.025	1.031
		NO_2_	0.002	1.002	0.029	1.000	1.004
27		UTCI	0.006	1.006	<0.0001	1.005	1.008
		PM_10_	0.001	1.001	0.002	1.000	1.001
		NO	0.028	1.028	<0.0001	1.025	1.031
		NO_2_	0.002	1.002	0.009	1.001	1.004
28	+64 years	Tair	0.003	1.003	0.006	1.001	1.005
		NO	0.023	1.023	<0.0001	1.019	1.027
		NO_2_	0.003	1.003	0.001	1.001	1.005
28		PET	0.002	1.002	0.050	1.000	1.004
		PM_10_	0.001	1.001	0.049	1.000	1.001
		NO	0.022	1.023	<0.0001	1.019	1.027
		NO_2_	0.004	1.004	<0.0001	1.002	1.006
29		UTCI	0.003	1.003	0.002	1.001	1.005
		NO	0.023	1.023	<0.0001	1.019	1.027
		NO_2_	0.003	1.003	0.001	1.001	1.005
30	Respiratory diseases	Tair	−0.019	0.981	<0.0001	0.977	0.985
		NO	0.015	1.016	<0.0001	1.008	1.023
		NO_2_	0.007	1.007	0.001	1.003	1.011
31		PET	−0.015	0.985	<0.0001	0.982	0.988
		NO	0.007	1.016	<0.0001	1.001	1.023
		NO_2_	0.153	1.007	<0.0001	1.003	1.011
32		UTCI	−0.016	0.984	<0.0001	0.981	0.988
		NO	0.016	1.006	<0.0001	1.009	1.024
		NO_2_	0.006	1.016	0.003	1.002	1.010

Abbreviations: CI, confidence interval; IRR, incidence rate ratio.

## Data Availability

The data presented in this study are available on reasonable request from the corresponding author.
